# Randomised phase III trial of irinotecan combined with cisplatin for advanced non-small-cell lung cancer

**DOI:** 10.1038/sj.bjc.6600725

**Published:** 2003-02-10

**Authors:** S Negoro, N Masuda, Y Takada, T Sugiura, S Kudoh, N Katakami, Y Ariyoshi, Y Ohashi, H Niitani, M Fukuoka

**Affiliations:** 1 Department of Clinical Oncology, Osaka City General Hospital, 2-13-22, Miyakojima-hondori, Miyakojima-ku, Osaka 534-0021, Japan; 2 2nd Department of Internal Medicine, Osaka Prefectural Habikino Hospital, Osaka 583-8588, Japan; 3 Department of Respiratory Diseases and Radiology, Hyogo Medical Center for Adults, Hyogo 673-0021, Japan; 4 Department of Respiratory Diseases, Aichi Cancer Center, Aichi 464-0021, Japan; 5 1st Department of Internal Medicine, Osaka City University Medical School, Osaka 545-8585, Japan; 6 Department of Pulmonary Diseases, Kobe City General Hospital, Hyogo 650-0046, Japan; 7 1st Department of Respiratory Diseases, Aichi Prefectural Hospital, Aichi 444-0011, Japan; 8 School of Health Sciences and Nursing, Faculty of Medicine, University of Tokyo, Tokyo 113-0033, Japan; 9 The Tokyo Cooperative Oncology Group, Tokyo 105-0012, Japan; 10 Department of Medical Oncology, Kinki University School of Medicine, Osaka 589-8511, Japan

**Keywords:** irinotecan, non-small-cell lung cancer, randomised phase III trial

## Abstract

To determine a standard combination chemotherapy for patients with advanced non-small-cell lung cancer (NSCLC), we conducted a phase III trial of irinotecan (CPT-11) to test the hypotheses that CPT-11+cisplatin is superior to cisplatin+vindesine and that CPT-11 monotherapy is not inferior to cisplatin+vindesine. A total of 398 patients with previously untreated NSCLC were randomised to receive cisplatin+CPT-11 (CPT-P), cisplatin+vindesine (VDS-P) or CPT-11 alone (CPT). In the CPT-P arm, CPT-11 60 mg m^−2^ was administered on days 1, 8 and 15, and cisplatin 80 mg m^−2^ was administered on day 1. In the VDS-P arm, cisplatin 80 mg m^−2^ was administered on day 1, and vindesine 3 mg m^−2^ was administered on days 1, 8 and 15. In the CPT arm, CPT-11 100 mg m^−2^ was administered on days 1, 8 and 15. The median survival time was 50.0 weeks for patients on CPT-P, 45.6 weeks for those on VDS-P and 46.0 weeks for those on CPT (*P*=0.115, CPT-P *vs* VDS-P; *P*=0.089, CPT *vs* VDS-P), and the hazard ratio was 0.85 (95% confidence interval (CI): 0.65–1.11) for CPT-P *vs* VDS-P and 0.83 (0.64–1.09) for CPT *vs* VDS-P. The response rate was 43.7% for patients on CPT-P, 31.7% for those on VDS-P and 20.5% for those on CPT. Major adverse reactions were grade 4 neutropenia observed in 37, 54 and 8% of the patients on CPT-P, VDS-P and CPT, respectively; and grades 3 and 4 diarrhoea observed in 12, 3 and 15% of the patients, respectively. CPT-P therapy produces comparable survival to VDS-P in patients with advanced NSCLC. CPT-11 monotherapy is not inferior to VDS-P in terms of survival. The CPT-11-containing regimen is one of the most efficacious and well tolerated in the treatment of advanced NSCLC.

In Japan, lung cancer is the leading cause of death among all the cancers, accounting for approximately 50 000 deaths annually (Statistics and Information Department, Minister's secretariat, Ministry of Health and Welfare, 2000). Non-small-cell lung cancer (NSCLC) accounts for more than 80% of primary lung cancers. Approximately two-thirds of NSCLC patients have advanced-stage cancer at presentation. The median survival time (MST) of the patients with advanced NSCLC, attained using the best available therapy (such as cisplatin-based chemotherapy), is typically 6–10 months, and most patients die of cancer within 1–2 years of diagnosis.

Irinotecan hydrochloride (CPT-11) is a water-soluble derivative of camptothecin, an alkaloid originally extracted from the Chinese tree *Camptotheca acuminata*. Differing from conventional antitumour drugs in mechanism of action, CPT-11 produces its effect by inhibiting the synthesis of DNA and RNA through inhibition of DNA topoisomerase I (Kawato *et al*, 1991). CPT-11 has shown strong antitumour activity as a single agent against a broad spectrum of experimental tumours as well as against human malignancies.

The phase I clinical trial of CPT-11, in which CPT-11 was administered weekly, showed that the dose-limiting adverse effects were leucopenia and diarrhoea, and the recommended dose for the phase II monotherapy trial was 100 mg m^−2^ week^−1^ (Negoro *et al*, 1991). In the phase II clinical trial of CPT-11 monotherapy for patients with untreated advanced NSCLC, the response rate was 31.9% (23 out of 72 cases) and the MST was 42 weeks (Fukuoka *et al*, 1992).

In preclinical studies, CPT-11 was confirmed to act synergistically with cisplatin (CDDP) (Kudoh *et al*, 1993). Since CDDP is active against NSCLC and forms part of the standard therapeutic armamentarium employed in the treatment of NSCLC, a phase I trial of CPT-11+CDDP was initiated in 1991 (Masuda *et al*, 1992). From this trial, ‘60 mg m^−2^ CPT-11 on days 1, 8 and 15, and 80 mg m^−2^ CDDP on day 1' were recommended as the optimal dose schedule. In 1992, a phase II trial of CPT-11+CDDP for untreated, stage IIIB or IV NSCLC was started employing this dose schedule; the response rate was 52% and the MST was 44 weeks (Masuda *et al*, 1998). These results suggested that CPT-11+CDDP could improve outcomes for patients with advanced NSCLC.

Previously, the National Cancer Institute of Canada (NCIC) Clinical Trials Group undertook a randomised study involving patients with NSCLC to determine whether there was a survival advantage for those patients treated with combination chemotherapy (vindesine (VDS)+CDDP or cyclophosphamide+doxorubicin+CDDP) over best supportive care (BSC). The results of that trial showed that the patients receiving VDS+CDDP had a statistically significant survival advantage over patients receiving BSC (Rapp *et al*, 1988). The VDS+CDDP regimen is one of the most widely used chemotherapeutic regimens for advanced NSCLC in Japan, and it was considered to be the most appropriate control regimen at the time when this study was initiated.

Based on the above results, we planned a phase III trial to compare CPT-11+CDDP and CPT-11 alone, with the control arm of VDS+CDDP, in order to elucidate the role of CPT-11 in advanced NSCLC.

## MATERIALS AND METHODS

### Eligibility criteria

Patients with histologically or cytologically confirmed and previously untreated NSCLC were enrolled into this trial. Patients with stage IIIB or IV cancer were eligible if they had measurable disease and a life expectancy of at least 3 months. Additional inclusion criteria were age (15–75 years old); a performance status (PS) of 0–2 on the Eastern Cooperative Oncology Group (ECOG) scale; and adequate functional indices for bone marrow (leucocyte count between ⩾4000 and <12 000/mm^−3^, platelet count ⩾100 000/mm^−3^, haemoglobin concentration ⩾9.5 g dl^−1^), liver (GOT and GPT <100 IU l^−1^, serum bilirubin ⩽1.5 mg dl^−1^), kidneys (serum creatinine ⩽the upper limit of normal) and lungs (*P*aO_2_; at rest ⩾8.0 kPa). Patients with other concurrent malignancies or a history of other malignancies, active infection, diarrhoea (watery stool), paralytic ileus, pulmonary fibrosis, pericardial effusion, considerable pleural effusion or ascites, uncontrolled diabetes mellitus or symptomatic metastasis to the brain, were excluded. Informed consent was obtained from each patient before enrollment. Each institutional review board for human experimentation approved the protocol of this study.

### Randomisation

**Figure 1:**
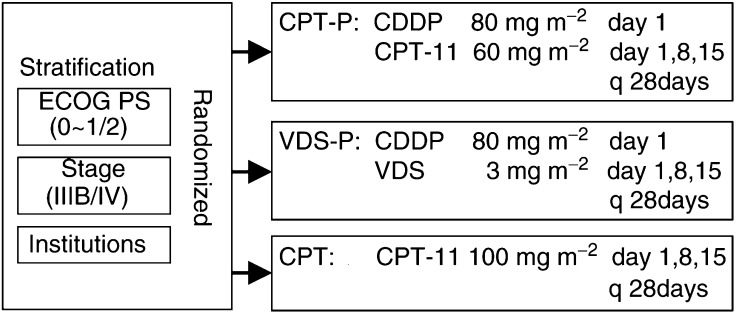
Study design. Patients enrolled were randomly allocated to receive CPT-P, VDS-P or CPT, after being stratified by PS, disease stage and institution. CPT-11=irinotecan; CDDP=cisplatin; VDS=vindesine.

Eligible patients were randomised to one of the three treatment arms by a centralised dynamic balancing method (a modified minimisation method) using stage (IIIB/IV), PS (0–1/2) and institution as balancing variables (Figure 1

).

### Treatment schedule

In the CPT-P arm, CPT-11 was given intravenously (i.v.) on days 1, 8 and 15 at a dose of 60 mg m^−2^, and CDDP was given i.v. on day 1 at a dose of 80 mg m^−2^. In the VDS-P arm, CDDP was given i.v. on day 1 at a dose of 80 mg m^−2^, and VDS was given i.v. on days 1, 8 and 15 at a dose of 3 mg m^−2^. In the CPT arm, CPT-11 was given i.v. on days 1, 8 and 15 at a dose of 100 mg m^−2^. In each arm, one course of treatment lasted 4 weeks and each course was repeated more than twice, until occurrence of unacceptable toxicity, disease progression, patient's refusal and investigator's medical decision.

CPT-11, diluted in ⩾500 ml of normal saline, was administered by i.v. infusion over 90 min. VDS was administered as an i.v. push with a running of 5–10 ml of normal saline. CDDP was administered i.v. as an undiluted solution and infused for a period of 60 min with more than 2600 ml of hydration and a diuretic before and after administration. To control CDDP-induced emesis, a 5-HT_3_ receptor antagonist was given before CDDP administration. In the CPT-P arm, CDDP was administered after the CPT-11 infusion.

### Dose modification

In all three arms, the dose of CPT-11 or VDS on day 8 or 15 was not given if the leucocyte count was less than 3000 mm^−3^, platelet count was less than 100 000 mm^−3^, body temperature was 37.5°C or higher, or diarrhoea was grade 1 (frequency increased to two times or more daily, abdominal pain rated as mild or severe, or watery stool) or higher on the ECOG scale.

In each arm, before the next course was started, the leucocyte count had to be 4000 mm^−3^ or more, platelet count had to be 100 000 mm^−3^ or more, serum creatinine concentration had to be normal, and diarrhoea and fever should have disappeared. If there was a delay greater than 4 weeks caused by persistent toxicity, patients in each arm were to be withdrawn from the study.

If during the previous course, the leucocyte count had been less than 1000 mm^−3^, platelet count less than 50 000 mm^−3^ or diarrhoea had been grade 2 (stool passage increased to four times or more daily, stool passage at night or moderate abdominal pain, watery stool) or higher on the ECOG scale, the dose of CPT-11 was reduced to 50 mg m^−2^ in the CPT-P arm, and to 80 mg m^−2^ in the CPT arm. If during the previous course the serum creatinine concentration was more than 1.5 times the upper normal limit, the dose of CDDP was reduced to 60 mg m^−2^ in the CPT-P arm and in the VDS-P arm.

### Evaluation

This trial was independently monitored and performed according to GCP rules.

The primary end point of this study was overall survival. Response rate, duration of response, time to disease progression and toxicity were all secondary end points.

The stage of the disease was determined based on a complete medical history and physical examination, routine chest radiographs, fibreoptic bronchoscopy, computed tomography of the head, chest and abdomen and bone scintigraphy. The staging estimates were made according to the international staging system (Mountain, 1986). Before the first course, the haemogram of each patient was determined and serum chemistry was used to check renal and hepatic functions, electrolytes and urinalysis. The haemogram was assessed at least twice a week, and serum chemistry, electrolytes, urinalysis and chest radiographs at least once weekly. Computed tomography of the chest was repeated monthly. Any other examination was carried out when any clinical sign of disease progression was observed. The eligibility, evaluability and response of each patient were reviewed extramurally. The assessment of antitumour effects and toxicity was based on the WHO criteria (World Health Organization, 1979), but diarrhoea was evaluated in accordance with the ECOG common toxicity criteria (Oken *et al*, 1982). The overall survival was defined as the time from randomisation to the time of death from any cause or to last follow-up (cutoff date: 23 January 2000). The duration of each response was defined as the time from the initial response to disease progression. The time to disease progression was defined as the time from the start of treatment to disease progression.

Health-related quality of life (QOL) was assessed experimentally with a self-administered QOL questionnaire for cancer patients treated with anticancer drugs (QOL-ACD) (Kurihara *et al*, 1999). The QOL-ACD was completed at baseline, every week during treatment and every month after treatment.

### Statistical analysis

The superiority of CPT-P to VDS-P and the noninferiority of CPT to VDS-P were evaluated in terms of survival benefit.

The sample size was calculated on the basis that MSTs were expected to be 44, 30 and 35 weeks for the CPT-P, VDS-P and CPT arms, respectively (Einhorn *et al*, 1986), and both the accrual and follow-up intervals were 2 years.

By using the Schoenfeld and Richter equation (1982), the least number of patients to provide the 80% power needed to confirm the superiority of a regimen was calculated to be 115 per treatment arm for a one-sided 2.5% significance level test. Furthermore, the least number of deaths to provide the 80% power needed to prove the noninferiority for the upper limit at a 95% CI for the hazard ratio of CPT compared with VDS-P was lower than the upper equivalence margin, 1.33, and was estimated to be 81 per treatment arm at the one-sided 2.5% level. The significance levels for both inferences were set at 2.5% to control the overall type I error rate, and the one-sided statistical approach was employed to keep the simplicity and structural consistency of the statistical inferences for two study hypotheses. Taking ineligible patients into account, the sample size was set at 130 per treatment arm.

Cumulative survival curves were constructed as time-to-event plots by the Kaplan–Meier method (1958). Differences between the curves were tested for significance using one-sided log-rank statistics, and were estimated for noninferiority using the hazard ratio produced by the Cox regression model (Cox and Oakes, 1984). Furthermore, Cox regression models were used to evaluate treatment effects on survival, with adjustment for well-known prognostic factors: stage, PS, gender, weight loss, albumin and LDH (Albain *et al*, 1991; Espinosa *et al*, 1995; Paesmans *et al*, 1995; Ray *et al*, 1998). Response rates and toxicities were compared using the *χ*^2^ test. Subgroup analysis for survival was conducted by stage.

## RESULTS

### Patient characteristics

From July 1995 to January 1998, 398 patients from 41 centres were entered into this study. Of the 398 patients randomised, 18 patients did not meet the eligibility criteria. The reasons for exclusion were: early stage (<IIIB) in five patients, previous treatment (OK-432, radiation therapy or surgery for local metastasis) in eight patients, other concurrent malignancies in two patients, age (>75 years) in one patient, presence of pericardial effusion in one patient, and leucocyte count (>12 000 mm^−3^) in one patient.

The patient characteristics at baseline are listed in Table 1

**Table 1:** Patient characteristics at baseline

	**CPT-P**	**VDS-P**	**CPT**
Patients entered	133	133	132
			
Eligible patients^a^	129	122	129
			
Gender			
Male	98	98	96
Female	31	24	33
			
Age (years)			
–49	10	19	22
50–59	36	29	30
60–69	58	51	52
70–	25	23	25
Median (range)	64 (36–75)	64 (35–75)	62 (35–75)
			
PS			
0–1	121	115	121
2	8	7	8
			
Stage			
IIIB	49	46	44
IV	80	76	85
			
Histology			
Adenocarcinoma	78	73	84
Squamous cell carcinoma	37	45	34
Others	14	4	11
			
Weight loss			
No	74	67	77
Less than 5%	10	14	9
5% or more	27	30	29
Unknown	18	11	14

a Patients who were used to evaluate survival.

CPT=irinotecan; P=cisplatin; VDS=vindesine; PS=performance status.

. The median age of the patients was 62–64 years and the range was 35–75 years. A total of 62% of the patients had adenocarcinoma, 31% had squamous cell carcinoma, 37% had stage IIIB disease and 94% of the patients had a good performance status (PS 0–1). The three treatment groups were well balanced for all baseline characteristics.

### Treatment administration

Three patients received no treatment (one patient in the CPT-P arm and two patients in the CPT arm), two patients in the VDS-P arm received vincristine (VCR) instead of VDS, one patient in the CPT-P arm exceeded the daily dose of CPT-11 and CDDP, and one patient in the CPT-P arm received CDDP on days 1 and 8. These seven patients were included in the survival analysis, but excluded from response and toxicity analysis. In all, 109 of the 126 patients on CPT-P (87%), 97 of the 120 patients on VDS-P (81%) and 101 of the 127 patients on CPT (80%) received more than two courses of treatment, and 22 patients (18%) on CPT-P, 15 patients (13%) on VDS-P and 20 patients (16%) on CPT received more than four courses of treatment. The median number of courses administered per patient was 3 for CPT-P and 2 for both VDS-P and CPT. The number of treatment courses varied from one to six in each arm. In the CPT-P, VDS-P and CPT arms, 23, 10 and 16 patients were withdrawn from the study because of toxicities (including duplications); 28, 44 and 51 patients because of disease progression; 37, 27 and 19 patients due to patient's refusal; 10, 19, and 22 patients because of aggravated clinical symptom; 50, 32 and 28 patients due to investigator's medical decision; and 6, 9 and 9 patients because of other reasons, respectively. Most patients could repeat treatment courses every 28 days irrespective of the arm.

The number of treatment courses in which CPT-11 or VDS was administered on days 1, 8 and 15 totaled 164 courses (50% of a total of 330 courses) for the CPT-P arm, 66 courses (24% of a total of 279 courses) for the VDS-P arm and 182 courses (62% of a total of 292 courses) for the CPT arm. Dose omissions on day 8 and/or day 15 were because of diarrhoea and/or leucopenia in the CPT-P arm and the CPT arm, and because of leucopenia in the VDS-P arm. The median dose intensity of CDDP was the same (20 mg m^−2^ week^−1^) for both the CPT-P and VDS-P arms. The median dose intensity of CPT-11 was 30 mg m^−2^ week^−1^ (67% of planned dose) for the CPT-P arm and 61.3 mg m^−2^ week^−1^ (82% of planned dose) for the CPT arm. The median dose intensity of VDS was 1.5 mg m^−2^ week^−1^ (67% of planned dose).

### Survival

**Figure 2:**
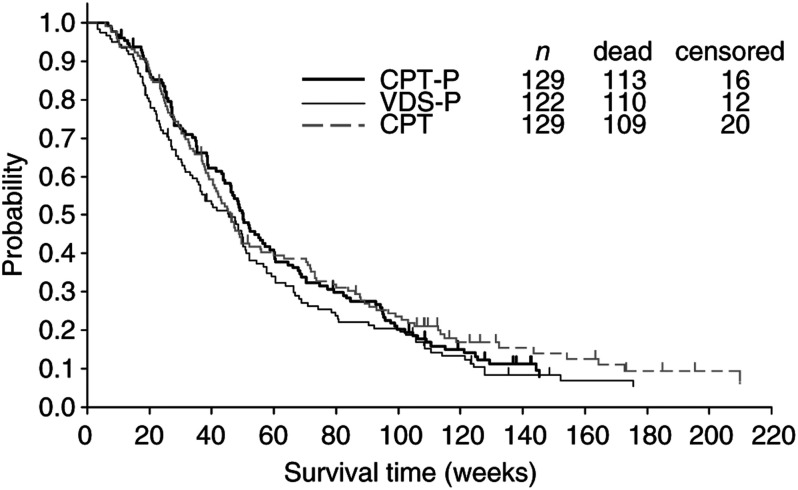
Survival of eligible patients. Survival time was calculated from the date patients were entered into this study. CPT=irinotecan; P=cisplatin; VDS=vindesine; *n*=number of eligible patients.

The survival curves for all eligible patients are shown in Figure 2

. The MST was 50.0 weeks for patients on CPT-P, 45.6 weeks for those on VDS-P and 46.0 weeks for those on CPT, and the 1- and 2-year survival rates were 46.5 and 19.4% for patients on CPT-P, 38.3 and 18.7% for those on VDS-P, and 41.8 and 21.9% for those on CPT. The one-sided log-rank test comparing the survival of patients who received CPT-P *vs* those treated with VDS-P yielded a *P*-value of 0.115, and when the survival of patients treated with CPT was compared with that of patients treated with VDS-P, the *P*-value was 0.089. The hazard ratio was 0.85 (95% CI: 0.65–1.11) for CPT-P *vs* VDS-P and 0.83 (95% CI: 0.64–1.09) for CPT *vs* VDS-P.

**Figure 3:**
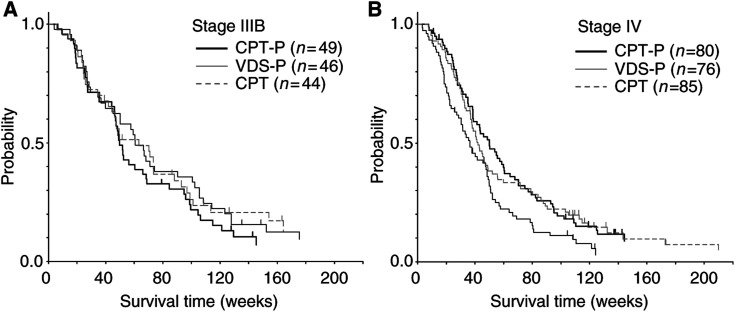
Survival of eligible patients: (**A**) stage IIIB, (**B**) stage IV. Survival time was calculated from the date patients were entered into this study. CPT=irinotecan; P=cisplatin; VDS=vindesine; *n*=number of eligible patients.

Subgroup analyses for survival were conducted using stage as one of the balancing variable factors (Figure 3

). Among patients with stage IIIB disease (Figure 3A), the MST was 49.7 weeks for those on CPT-P, 60.6 weeks for those on VDS-P and 63.3 weeks for those on CPT. The hazard ratio was 1.24 (95% CI: 0.81–1.91, one-sided, log-rank test: *P*=0.838) for CPT-P *vs* VDS-P and 0.99 (95% CI: 0.63–1.56, one-sided, log-rank test: *P*=0.483) for CPT *vs* VDS-P.

Among patients with stage IV disease (Figure 3B), the MST was 50.0 weeks for those on CPT-P, 36.4 weeks for those on VDS-P and 42.1 weeks for those on CPT. The hazard ratio was 0.64 (95% CI: 0.46–0.89, one-sided, log-rank test: *P*=0.004) for CPT-P *vs* VDS-P and 0.70 (95% CI: 0.50–0.98, one-sided, log-rank test: *P*=0.018) for CPT *vs* VDS-P.

The Cox proportional hazards model was used to adjust for and determine the impact of prognostic factors and treatment on survival (Table 2

**Table 2:** Cox proportional hazards model

**Variable**	**Hazard ratio**	**95% CI**	***P*-value**
Regimen			
CPT-P/VDS-P	0.80	0.60–1.07	0.1341
CPT/VDS-P	0.88	0.66–1.16	0.3557
Stage (IV/IIIB)	1.70	1.32–2.19	0.0001
PS (2/1/0)	1.40	1.08–1.80	0.0098
Gender (male/female)	1.29	0.96–1.73	0.0922
Weight loss (⩾5%/<5%)	2.00	1.53–2.60	0.0001
Albumin (<4.0 g dl^−1^/⩾4.0 g dl^−1^)	1.53	1.17–1.98	0.0016
LDH (⩾400 IU l^−1^/<400 IU l^−1^)	1.54	1.17–2.04	0.0021

CI=confidence interval; CPT=irinotecan; P=cisplatin; VDS=vindesine; PS=performance status; LDH=lactic acid dehydrogenase.

). Predictive factors for improved survival included early clinical stage, no weight loss, normal LDH, normal albumin and better PS, each of which was significantly associated with longer survival.

### Response

Objective response data are listed in Table 3

**Table 3:** Objective response

	***n***	**CR**	**PR**	**NC**	**PD**	**NE**	**ORR (%)**
CPT-P	126	3	52	51	17	3	43.7
VDS-P	120	1	37	54	25	3	31.7
CPT	127	1	25	66	33	2	20.5

*n*=number of assessable patients, CR=complete response, PR=partial response, NC=no change, PD=progressive disease, NE=not evaluable, ORR=objective response rate, CPT=irinotecan, P=cisplatin, VDS=vindesine.

. The overall response rate was 43.7% for patients on CPT-P, 31.7% for those on VDS-P and 20.5% for those on CPT (two-sided, *χ*^2^ test: *P*<0.001).

The median duration of response for responders was 141 days for patients on CPT-P, 121 days for those on VDS-P, and 117 days for those on CPT (two-sided, log-rank test: *P*=0.601). The median time to progression for the patients included in the efficacy analysis was 148 days for patients on CPT-P, 117 days for those on VDS-P and 100 days for those on CPT (two-sided, log-rank test: *P*=0.091).

### Toxicity

Major adverse reactions are listed in Table 4

**Table 4:** Major adverse reactions

	**CPT-P**	**VDS-P**	**CPT**
	**(*n*=126)**	**(*n*=120)**	**(*n*=127)**
Leucopenia (grade 4)	8 (6%)	4 (3%)	2 (2%)
Neutropenia (grade 4)	46 (37%)	65 (54%)	10 (8%)
– Febrile neutropenia	13 (10%)	13 (11%)	8 (6%)
Thrombocytopenia (grade 4)	4 (3%)	1 (1%)	0 (0%)
Anaemia (grade 4)	2 (2%)	1 (1%)	1 (1%)
GOT↑ (grades 3 and 4)	0 (0%)	1 (1%)	0 (0%)
GPT↑ (grades 3 and 4)	1 (1%)	1 (1%)	0 (0%)
BUN↑ (grades 3 and 4)	2 (2%)	2 (2%)	0 (0%)
Creatinine↑ (grades 3 and 4)	0 (0%)	1 (1%)	0 (0%)
Diarrhoea (grades 3 and 4)	15 (12%)	4 (3%)	19 (15%)
Nausea/vomiting (grades 3 and 4)	41 (33%)	27 (23%)	12 (9%)
Infection (grades 3 and 4)	4 (3%)	1 (1%)	6 (5%)

CPT=irinotecan; P=cisplatin; VDS=vindesine; GOT=glutamic oxaloacetic transaminase; GPT=glutamic pyruvic transaminase; BUN=blood urea nitrogen.

. Grade 4 neutropenia was observed significantly more frequently in the VDS-P arm than in the CPT-P and CPT arms, both of which contain CPT-11 (*P* < 0.001). Grade 4 thrombocytopenia was more frequent in the CPT-P arm, but there were no thrombocytopenia-related complications.

Diarrhoea was the main nonhaematological sign of toxicity of the regimens containing CPT-11 (CPT-P and CPT); these two regimens were associated with a significantly higher occurrence of grades 3 and 4 diarrhoea as compared with the VDS-P regimen (*P*=0.008). The regimens containing CDDP (CPT-P, VDS-P) were associated with a significantly higher occurrence of grade 3 nausea/vomiting as compared with the CPT alone regimen (*P*=0.001). Peripheral neurological symptoms were observed significantly more frequently in the VDS-P group than in the other two groups (*P*<0.001).

Two patients died of causes whose relation to the chemotherapy treatment could not be completely ruled out. A patient who had received VDS-P died because of bleeding from the digestive tract after recovery from myelosuppression. Another patient who had received CPT died owing to subsequent infection associated with severe diarrhoea after recovery from myelosuppression. There were no cases of treatment-related death in the CPT-P arm.

### QOL

A total of 82 patients in the CPT-P arm, 65 patients in the VDS-P arm and 74 patients in the CPT arm completed at least one QOL questionnaire. Compliance with the QOL questionnaire was 65% on CPT-P, 54% on VDS-P and 58% on CPT. We could not adequately evaluate QOL for the three arms because compliance with the QOL questionnaire was low. Merely crude analysis suggested no difference among the three arms in terms of QOL.

### Second-line treatment

Among patients with stage IIIB disease, 45% on CPT-P, 61% on VDS-P and 59% on CPT were subsequently treated with thoracic irradiation.

Among patients with stage IV disease, 25% on CPT-P, 26% on VDS-P and 40% on CPT were subsequently treated with other chemotherapeutic regimens. In particular, seven patients on CPT-P, nine patients on VDS-P and 10 patients on CPT were subsequently treated with docetaxel (DTX) monotherapy or DTX-containing regimens.

## DISCUSSION

Since 1990, newer agents such as CPT-11, paclitaxel (PTX), DTX, gemcitabine (GEM) and vinorelbine (VNB) have shown response rates of 20% or more when used alone in previously untreated NSCLC (Bunn and Kelly, 1998).

Except for CPT-11, all four chemotherapeutic agents have been examined in many phase III trials in patients with advanced NSCLC and their roles have been mostly elucidated. No trial has been conducted, however, in order to establish the role of CPT-11 in advanced NSCLC. The present clinical trial was the first phase III study of CPT-11 in patients with advanced NSCLC. We planned this phase III trial in order to verify (1) whether CPT-P significantly prolongs the survival time and (2) whether CPT alone is not inferior in terms of the survival time, as compared with VDS-P, one of the most commonly used chemotherapeutic regimen that has been used in advanced NSCLC.

The first results showed that CPT-P did not significantly prolong the survival time compared with VDS-P. Previous studies of PTX (Belani *et al*, 1998; Giaccone *et al*, 1998; Bonomi *et al*, 2000), DTX (Kubota *et al*, 2002), GEM (Crino *et al*, 1999) and VNB (Le Chevalier *et al*, 1994; Martoni *et al*, 1998) were for comparison of a platinum compound plus a new agent *vs* an old platinum-based chemotherapy. Only the studies by Le Chevalier *et al* (1994) and Kubota *et al* (2002) reported significant differences in terms of survival based on the analysis scheduled in the protocol. Similar to the present trial, there was no significant prolongation of the survival time in other studies. There were meta-analyses of these results that reported the superiority of the regimen of platinum compounds plus a new agent (Baggstrom *et al*, 2002; Yana *et al*, 2002).

The second results showed that CPT-11 alone was not inferior to VDS-P in terms of the survival time. In a study of Le Chevalier *et al* (1994) that compared three groups (VNB and CDDP *vs* VDS and CDDP *vs* VNB alone), similar to the present trial, the survival was also similar between VNB alone and VDS-P. Toxicity on CPT alone was mild except for slightly frequent diarrhoea. Based on these results, chemotherapy with the new single agent may be considered for patients who are unable to receive CDDP-containing regimens because it can produce the similar survival to conventional platinum-based combinations, and toxicity is relatively mild.

In the subgroup analyses according to the stage, in stage IV patients, the MST was 50.0 weeks on CPT-P and 36.4 weeks on VDS-P, with the CPT-P being superior to the VDS-P with respect to survival prolongation (one-sided, log-rank test: *P*=0.004). CPT-11 alone also significantly prolonged the survival time in patients with stage IV NSCLC compared with VDS-P. Among stage IV patients, there was no bias in terms of background factors including PS (the ratio of PS 0–1 patients was 96% on CPT-P, 92% on VDS-P and 94% on CPT), gender (male ratio was 69% on CPT-P, 79% on VDS-P and 68% on CPT) and the rate of weight loss at baseline (the ratio of patients without weight loss was 63% on CPT-P, 58% on VDS-P and 55% on CPT). There was no bias in the content of second-line treatment among the treatment arms. These are just the results of the subgroup analyses and their significance should be shown in another research.

The results of subgroup analyses differed between stage IIIB and stage IV patients. Between-arm differences in the ratio of patients receiving second-line chest irradiation might, in part, lead to such differences. In the present trial, there was no eligibility criterion to restrict stage IIIB patients for whom thoracic irradiation was indicated. Therefore, the possibility could not be ruled out that the present trial included patients who were not suitable for research purely comparing chemotherapeutic agents as in the case of this trial. This might be one reason for different results between stages IIIB and IV patients in the subgroup analyses. In future research, the inclusion of stage IIIB patients should be limited to patients for whom definitive thoracic radiation is not indicated.

Comparing survival between CPT-P and CPT-11 alone, although this was not planned, no significant difference was shown between the two arms (one sided, log-rank test: *P*=0.587). The hazard ratio for CPT *vs* CPT-P was 1.03 (95% CI: 0.79–1.34).

The overall response rate in this study was 43.7% on CPT-P, 31.7% on VDS-P and 20.5% on CPT (two-sided, *χ*^2^ test: *P*<0.001). This significant difference in the response rate did not lead to significant differences in the survival time. Similar results have been frequently reported in comparative studies of chemotherapeutic agents in advanced NSCLC. Various known and unknown prognostic factors were involved in this result. Of them, the major factor is that complete response (CR) is obtained only in small numbers of NSCLC patients receiving chemotherapy. In the present trial, the CR rate was just 2.4% (three out of 126) in the CPT-P arm, 0.8% (one out of 120) in the VDS-P arm and 0.8% (one out of 127) in the CPT arm.

The present trial allowed including patients with PS 2. This resulted in the inclusion of approximately 6% of PS 2 patients in each treatment arm. In a large-scale ECOG study (E1594) by Schiller *et al* (2002), the accrual of patients with PS 2 was discontinued owing to the high rate of serious adverse events. This fact was disclosed in May 1999 (Johnson *et al*, 1999), and our trial completed patient registration before this time, so it was impossible to exclude PS 2 patients. Inclusion of PS 2 patients had no significant impact on the results of the present trial because the number of such patients was small and they were distributed evenly to the three arms according to baseline demographic factors.

Neutropenia and neurotoxicity were more frequent in the non-CPT-containing VDS-P arm, than in the CPT-containing CPT-P and CPT arms. Nausea/vomiting was more frequent in the CDDP-containing CPT-P and VDS-P arms, than in the non-CDDP-containing CPT arm. Diarrhoea was more frequent in the CPT-containing arm than in the VDS-P arm. In the present trial, there were no deaths associated with the treatment in the CPT-P arm, but one patient in the CPT arm developed diarrhoea after recovery from myelosuppression and subsequently died from infection. In this patient, it was not clear whether the source of infection was the bowel or the tip of the catheter. In 10 patients treated with the regimen containing CPT-11 who developed grade 3 or higher infection, seven patients developed diarrhoea coincidentally with myelosuppression, which suggested enteritis as the source of infection. Infection was avoided in many patients by taking proactive measures against aggravation of diarrhoea and taking early anti-infection measures.

In conclusion, the response rate was significantly higher in patients with CPT-P therapy compared with VDS-P, and the survival time and toxicity were comparable, although this therapy failed to demonstrate significant survival prolongation. Based on these results, a large-scale randomised phase III trial comparing CPT-P as the control regimen with three new drugs (NVB, PTX, GEM) containing platinum-based doublets is currently underway in Japan. CPT-11 monotherapy produced survival result that was not inferior to that obtained with VDS-P, but produced less toxicity. Further studies are necessary to determine the significance of CPT-11 monotherapy in the treatment of NSCLC. This randomised phase III trial has demonstrated that the regimen containing CPT-11 is one of the most active and well tolerated in the treatment of advanced NSCLC.

## APPENDIX

*The following members and institutions participated in this study*: S Nakamura, 4th Department of Internal Medicine, Osaka Medical Center for Cancer and Cardiovascular Diseases, Osaka; M Kawahara, Department of Internal Medicine, National Kinki Central Hospital for Chest Diseases, Osaka; S Yokota, Department of Internal Medicine, Toneyama National Hospital, Osaka; T Nakano, 3rd Department of Internal Medicine, Hyogo College of Medicine, Hyogo; N Hara, Research Institute for Diseases of the Chest, Research Institute for Diseases of the Chest, Faculty of Medicine, Kyushu University, Fukuoka; H Senba, Division of Respiratory Diseases, Kumamoto Regional Medical Care Center, Kumamoto; I Ohata, Department of Respiratory Diseases, Shimonoseki Kosei Hospital, Yamaguchi; T Sawa, Division of Respiratory Medicine, Gifu Municipal Hospital, Gifu; K Bando, Department of Respiratory Medicine, Saiseikai Nakatsu Hospital, Osaka; M Takada, Department of Respiratory Diseases, Izumisano Municipal Hospital, Osaka; Y Tanio, Department of Internal Medicine, Osaka Prefectural General Hospital, Osaka; S Sakai, Department of Respiratory Diseases, Japanese Red Cross Nagoya First Hospital, Aichi; M Oka, 2nd Department of Internal Medicine, School of Medicine, Nagasaki University, Nagasaki; T Igarashi, 2nd Department of Internal Medicine, Osaka Posts and Telecommunications Hospital, Osaka; H Nishiyama, Department of Respirology Medicine, Japanese Red Cross Society Wakayama Medical Center, Wakayama; T Kamei, Department of Internal Medicine, Kagawa Prefectural Center Hospital, Kagawa; S Hayashi, Department of Internal Medicine, Saga Medical School, Saga; J Araki, Department of Internal Medicine, Sasebo City General Hospital, Nagasaki; M Fujimura, 3rd Department of Internal Medicine, School of Medicine, Kanazawa University, Ishikawa; T Hoso, Department of Respiratory Medicine, Wakayama Rosai Hospital, Wakayama; H Yanagawa, 3rd Department of Internal Medicine, School of Medicine, The University of Tokushima, Tokushima; T Isobe, 2nd Department of Internal Medicine, Hiroshima University School of Medicine, Hiroshima; T Tsuya, Department of Pulmonary Diseases, Chugoku Rosai Hospital, Hiroshima; A Motohiro, Department of Surgery, National Minamifukuoka Chest Hospital, Fukuoka; H Yamamoto, Department of Pulmonary Diseases, Aso Cement Iizuka Hospital, Fukuoka; M Ichiki, 1st Department of Internal Medicine, Kurume University, School of Medicine, Fukuoka; S Hayasaka, Department of Internal Medicine, Kumamoto Chuo Hospital, Kumamoto; T Kanda, Department of Internal Medicine, Nagasaki Municipal Citizens Hospital, Nagasaki; H Fujiwara, 2nd Department of Internal Medicine, Gifu University School of Medicine, Gifu; M Kono, Department of Radiology, Kobe University School of Medicine, Hyogo; K Daido, Department of Respiratory Diseases, Hiroshima Red Cross Hospital, Hiroshima; Y Ichinose, Department of Chest Surgery, National Kyushu Cancer Center, Fukuoka; Y Yamaji, Respiratory Division, Mitoyo General Hospital, Kagawa.
